# Decoding Owl Calls: Refining Occupancy Inference From Passive Acoustic Monitoring

**DOI:** 10.1002/ece3.72255

**Published:** 2025-10-09

**Authors:** Natalie M. Rugg, Cara L. Appel, Julianna M. A. Jenkins, Chris McCafferty, Taal Levi, Damon B. Lesmeister

**Affiliations:** ^1^ Pacific Northwest Research Station USDA Forest Service Corvallis Oregon USA; ^2^ Department of Fisheries, Wildlife, and Conservation Sciences Oregon State University Corvallis Oregon USA

**Keywords:** interpretation, northern spotted owl, passive acoustic monitoring, space use, species detection, territorial behavior, vocalization patterns

## Abstract

Many territorial species use vocalizations as a primary form of territory defense, and the areas actively defended do not necessarily correspond with an individual's home range. As passive acoustic monitoring becomes a primary population assessment method for soniferous species, often in combination with occupancy modeling, effective conservation will require more detailed information on species‐specific space use to refine interpretation. Northern spotted owls ( *Strix occidentalis caurina* ) actively vocalize during the breeding season, but interpreting acoustic data is complicated by variable detectability—particularly for females—and interference from competitive barred owls (*Strix varia*). Using a dense network of autonomous recording units deployed 0–3 km from known northern spotted owl activity centers, we quantified vocal activity by sex, reproductive status, landscape features, and intensity of barred owl vocalizations. Additionally, we compared detections from overlapping regional monitoring sites to refine our understanding of detectability. Male territorial calls were detected more frequently and consistently than female territorial calls. Female calls were infrequent and restricted to the activity center and immediately adjacent areas, especially when nesting. Vocal space use areas were similar in size but smaller than published home ranges, reinforcing that territorial calls represent high‐use areas, not full spatial use. We propose a detection‐based spectrum of weeks with detection for inferring occupancy that accounts for calling rate, caller sex, and project objectives. Stricter thresholds can minimize false positives in population assessments, while inclusive thresholds reduce false negatives when used to determine habitat protection. Our results support nuanced, objective‐based thresholds for interpreting northern spotted owl detections from passive acoustic monitoring. This approach balances accuracy with conservation risk tolerance, elucidating that acoustically inferred territory does not reflect total landscape used. By clarifying vocal behavior, this study advances the application of passive acoustic monitoring for habitat management and occupancy estimation amid intense interspecies pressures and ongoing landscape change.

## Graphical abstract

Effective conservation often requires accurate occupancy and space use information, especially if passive acoustic monitoring becomes a primary population assessment tool. Using a dense array of autonomous recording units, we quantified northern spotted owl ( *Strix occidentalis caurina* ) vocal activity by sex, reproductive status, and ecological context. Vocal space use areas were similar in size to core use areas but smaller than published home ranges, supporting nuanced interpretation of detections to distinguish true occupancy from broader landscape use from passive acoustic monitoring.

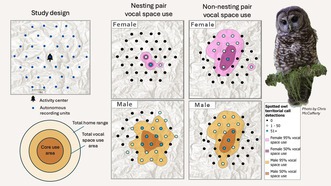

## 1 Introduction

Accurately monitoring wildlife space use and occupancy is fundamental to conservation, yet remains challenging for wide‐ranging, cryptic species. Many territorial animals defend key resources through conspicuous signals, including vocalizations, that offer opportunities for efficient, non‐invasive monitoring (Sugai et al. 2018). Passive acoustic monitoring has emerged as a scalable tool for detecting species presence and behavior, with growing applications across taxa and landscapes (Ross et al. 2023). However, interpreting acoustic detections requires careful consideration of sampling design, species ecology, vocal behavior, and imperfect detection, especially when management decisions hinge on distinguishing true occupancy from transient use (Berigan et al. 2019).

Territoriality, habitat specialization, and competition all shape patterns of space use, influencing both the likelihood of detection and the ecological meaning of observed patterns (Ross et al. 2023). The northern spotted owl ( *Strix occidentalis caurina* ; hereafter “spotted owl”) exemplifies these challenges. As an old‐forest obligate in the Pacific Northwest, USA, its status has profound implications for land management, with site occupancy directly influencing timber harvest and habitat protection on public and private lands (Franklin et al. 2021). Due to rapid habitat loss and associated population decline, the spotted owl was listed as threatened under the Endangered Species Act in 1990 and now warrants uplifting to endangered status (US Fish and Wildlife Service 2020; Franklin et al. 2021). As a result, the spotted owl has been the focus of intensive monitoring for over three decades (Franklin et al. 2021; Lesmeister et al. 2018). Surveys have transitioned from mark‐resight studies to passive acoustic monitoring, with detection/non‐detection data analyzed in an occupancy framework (Lesmeister and Jenkins 2022). While passive acoustics provide unprecedented spatial and temporal coverage, translating detections into actionable information remains a major challenge because detections indicate vocal activity rather than precise locations or demographic states.

Compounding these challenges, the spotted owl faces escalating competition from the barred owl ( *Strix varia* ), whose range expansion across western North America was facilitated by landscape changes (Livezey 2009). Larger size, broader diet, and interspecific competition, compounded by higher densities of barred owls in landscapes where they have become established, give them a competitive advantage over spotted owls (Wiens et al. 2014, 2021). Both species rely heavily on vocalizations for territory defense and pair communication (Forsman et al. 1984; Odom and Mennill 2010), and spotted owls may call less in the presence of barred owls, depending on ecological context (Appel et al. 2023; Crozier et al. 2006; Duchac et al. 2020). Sex‐specific differences in spotted owl calling rates further necessitate a nuanced interpretation of acoustic data (Appel et al. 2023).

Spotted owls are non‐migratory, year‐round territorial residents with high mate and site fidelity (Forsman et al. 1984). During the breeding season, home ranges contract, and pair space use becomes more concentrated—typically localized near a nest or primary roost area since spotted owls are central‐place foragers (Hamer et al. 2007). Among the spotted owl's vocal repertoire, the four‐note territorial call is the most distinct and strongly associated with territory defense (Forsman et al. 1984). Territorial vocalizations are energetically costly and carry risks; for example, exposure to predators (Ophir et al. 2010) such as great‐horned owls ( *Bubo virginianus* ), thought to be a primary predator of spotted owls (Forsman et al. 1984; Paton et al. 1991; Wiens et al. 2021). While these calls indicate territory defense, the locations of vocalizations do not necessarily reflect the full extent of the home range (Anich et al. 2009; Chronister et al. 2025; Reid et al. 2022). Home ranges of neighboring spotted owls often overlap, likely reflecting foraging areas too large to defend, suggesting that defended territories are not synonymous with home ranges (Hamer et al. 2007). Understanding the spatial relationship between calling activity and broader space use is critical for accurate occupancy inference and for identifying areas for habitat protection from landscape management.

In occupancy models, closure assumptions require that sites do not change occupancy status between repeated survey occasions within a season. Weekly survey intervals over 5–8 weeks are commonly used for modeling spotted owl detections (Appel et al. 2023; Lesmeister et al. 2021). If individuals move among sites during the survey season, *occupancy* is indistinguishable from *landscape use*. Distinguishing occupancy from landscape use has key benefits for both long‐term monitoring and forest management. Violations of occupancy assumptions can lead to overestimation of populations and therefore underestimation of population changes (Berigan et al. 2019; Lesmeister et al. 2021). Measures of abundance and the location of nest sites can be important information for management and conservation (Glenn et al. 2017). Identifying true occupancy extends the use of passive acoustic monitoring beyond detection/non‐detection, one step closer to abundance estimation and nest location identification. During the breeding season, activity center fidelity is high—particularly for females—making closure more plausible (Forsman et al. 1984). When male and female detections occur within a short window and spatial extent, pair occupancy can be inferred (Appel et al. 2023; Lint et al. 1999). However, due to the infrequency of female vocalizations, detecting only male territorial vocalizations may reflect an unobserved paired status. Appel et al. (2023) estimated true spotted owl pair occupancy to be 1.3–4.1 times greater than observed, underscoring the need to account for imperfect detection.

Spotted owl calling rates and site use are expected to decline with distance from the activity center (Duchac et al. 2020; Forsman et al. 1984; Wiens et al. 2014), allowing inference on pair status and territory boundaries. However, the precise relationship between calling rates and distance is unclear and further obfuscated by ecological context (e.g., breeding vs. non‐breeding years). We investigated how resident spotted owl vocal detectability and space use vary with distance from known activity centers using a large array of autonomous recording units (ARUs) spaced 0–3 km from known activity centers. We quantified calling rates, caller sex, and spatial distribution of calls. We compared vocal space use areas to published home range estimates (see Glenn et al. 2004; Hamer et al. 2007) and assessed variation by sex, nesting status, and barred owl vocalization intensity. Our results aim to improve discrimination between occupancy and broader landscape use from limited acoustic detections, expanding the application of passive acoustic monitoring and reducing uncertainty in interpretation.

## 2 Methods

### 2.1 Data Collection

We studied spotted owl activity centers in Oregon and Washington, USA, across three USDA Forest Service National Forests: Umpqua, Siuslaw, and Okanogan‐Wenatchee. Early in the 2021 and 2022 breeding seasons (March–April), we identified activity centers by visiting previously documented nest sites. At eight sites with at least one territorial individual, we assessed pair and nesting status following standardized protocols to assign each an activity center, which is either a primary roost or nest site from which foraging areas extend (Lint et al. 1999). Sites varied in reproductive status across years.

**FIGURE 1:**
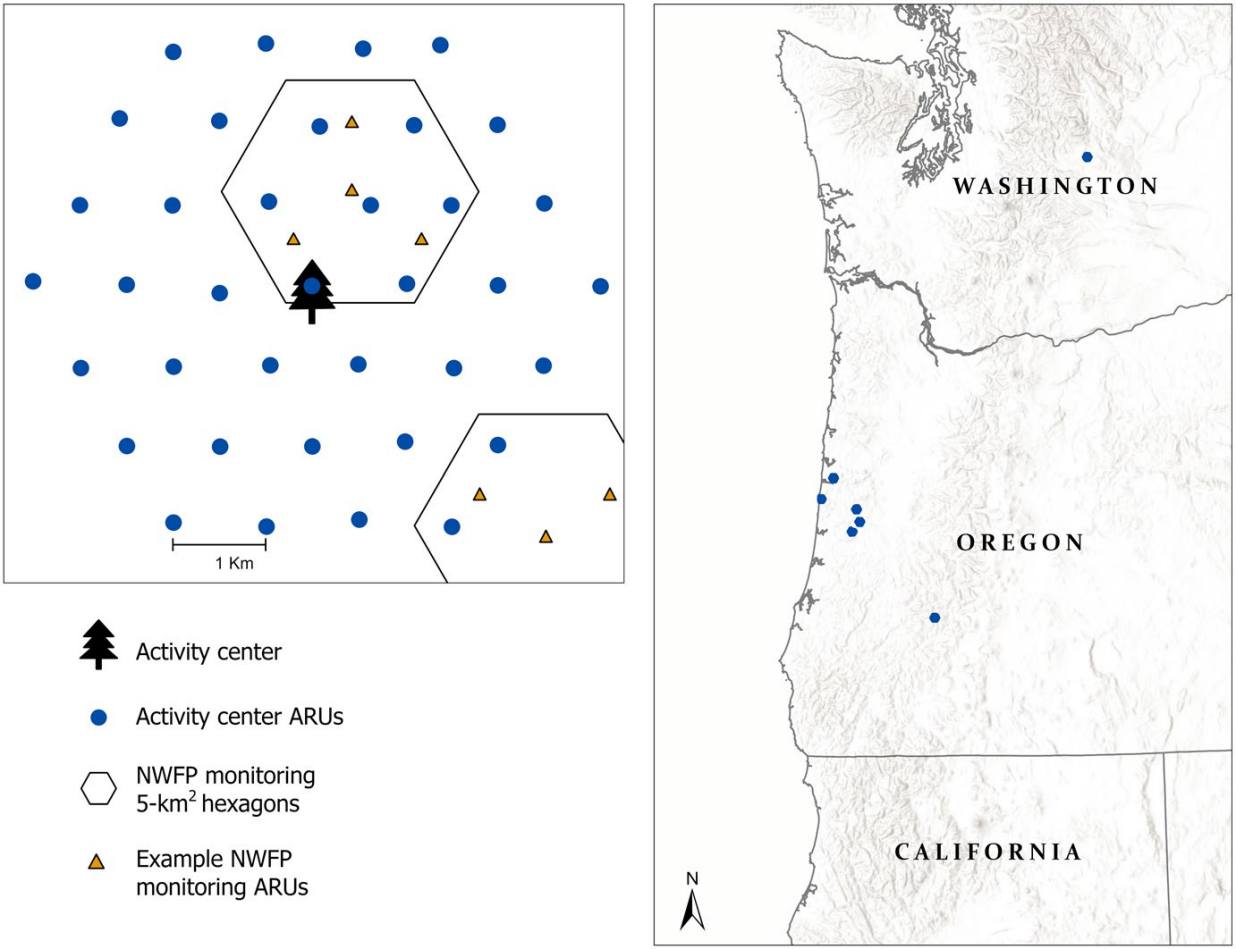
Study areas in western Oregon and Washington, USA, and sample design including autonomous recording units (ARUs) deployed 1 km apart in hexagonal arrays extending up to 3‐km radii from eight northern spotted owl activity centers. Also shown are randomly selected 5‐km^2^ Northwest Forest Plan (NWFP) spotted owl monitoring program hexagons, each containing four ARUs.

Shortly after locating territorial owls, we deployed ARUs (Song Meter Mini, Wildlife Acoustics, Maynard, MA, USA) in hexagonal grids extending in 3‐km radii from each activity center, with ~37 stations per site (Figure 1). Since median nearest‐neighbor distances of spotted owl territories can range up to 1.75 km (Dugger et al. 2016), we are confident the ARU array would capture all territorial vocal activity of the focal individuals. ARUs were placed ~1 km apart on mid‐to‐upper slopes, adjusted to minimize background noise (> 50 m from roads and creeks; Lesmeister et al. 2022) and account for land ownership boundaries. While spotted owl territorial calls can carry up to 1 km under ideal conditions (Forsman et al. 1984), the typical effective recording distance is ≤ 250 m due to environmental attenuation (Hane et al. 2022).

ARUs recorded for ~4–5 months (April–September) and programmed to capture 2.5 h after sunset, 2.5 h before sunrise, and 10 min on the hour overnight to balance battery life, data storage, and high detection probability of spotted owls and barred owls (Duchac et al. 2020). Additionally, we compiled and compared data from ten passive acoustic survey areas deployed by the Northwest Forest Plan (NWFP) effectiveness monitoring program that by chance overlapped seven of our array areas (Lesmeister and Jenkins 2022). The NWFP monitoring program randomly selects 5‐km^2^ hexagonal cells from a regional grid of federal forest‐capable lands, each sampled with four ARUs deployed for ≥ 6 weeks between March and July and recording on a similar schedule for ~10 h per day (Lesmeister et al. 2021). This study qualitatively compared spotted owl detection rates by sex from NWFP monitoring hexagons to the vocal space use of focal owls to improve inference for surveys using the NWFP protocol.

### 2.2 Acoustic Data Processing

We processed all audio using a convolutional neural network (PNW‐Cnet v4) developed to support the NWFP monitoring program (Ruff et al. 2023). The workflow includes segmenting recordings into 12‐s clips and using PNW‐Cnet to assign classification scores (0–1) for each sound class (Ruff et al. 2021). We manually validated all apparent spotted owl territorial calls with classification scores ≥ 0.25 to maximize recall and classified detections by sex based on call frequency and duration (Dale et al. 2022). To avoid confounding caused by non‐focal owl calling, we manually identified spotted owl calls that could potentially be from non‐focal individuals by examining unique characteristics of calls (e.g., Kuntz and Stacey 1997) and censored these calls from analyses. We created three classes for caller sex: female, male, or unknown sex. Only territorial calls were used in analyses, which were typically “four‐note” calls (Forsman et al. 1984), but also included common variations of three‐note or five‐note calls.

To avoid confounding by call‐back surveys conducted for demographic monitoring that occurred concurrently with our study (e.g., Dugger et al. 2023), we removed detections suspected of resulting from call‐back surveys within 1.6 km of such surveys for activity center arrays or within the same hexagon for NWFP monitoring and within the same night as the survey. We used survey dates, times, and locations reported by call‐back surveyors and auditory cues surrounding spotted owl detections (e.g., survey tones, a series of pure tones at 0.5, 1.5, and 1.0 kHz) to confirm the occurrence of call‐back surveys. We then constructed weekly detection histories per ARU recording station, summarizing detections separately for female, male, and any sex (total calls, including unknown sex) territorial calls.

### 2.3 Analyses

This study used several model types to characterize and estimate the spatial distribution of spotted owl vocal activity. We first visualized the spatial intensity of male, female, and any‐sex territorial calls using 50% and 95% kernel density utilization distributions (UDs; Worton 1995). We used the number of days with detections at ARU stations to fit UDs with the *adehabitatHR* package in R with the ad‐hoc smoothing parameter and a 500‐m grid cell size (Calenge 2024). We chose generic settings to reflect the 1‐km distance between ARU stations and the caller location uncertainty from ARU station locations. Our UDs reflected spatial vocal activity distributions at the individual or pair level for spotted owls and at the species level for barred owls, as we did not attempt individual identification of barred owls. We created UDs for barred owl vocalizations to illustrate the area of coverage and intensity of barred owl species‐level calling activity.

We fit separate quasibinomial generalized linear models (GLMs) in program R with lme4 (R version 4.3.0; Bates et al. 2015) to model total spotted owl calling rates and sex‐specific calling rates on each ARU station, measured as the proportion of survey weeks with confirmed detections. The base model included distance to the spotted owl activity center (dist), and we considered additional models that tested for effects of spotted owl nesting status (nested), total apparent barred owl detections (bo_total), and proportion of weeks with a barred owl detection (bo_prop). This resulted in four models for each of any sex calls, female calls, and male calls. Given the high number of barred owl detections (e.g., 163,531 vs. 25,231 spotted owl calls) and higher model precision for barred owl classes (e.g., 0.97 vs. 0.84 for spotted owl; Ruff et al. 2023), we did not manually review all barred owl detections. Instead, we manually confirmed the presence of barred owls at each ARU station, then adjusted apparent barred owl call counts (classification score ≥ 0.95) by PNW‐Cnet v4 precision estimates for each call type to estimate the number of barred owl calls in each week at each ARU station.

We fit single‐season, single‐species occupancy models for male, female, and any sex spotted owl calls in the R package *unmarked* (Kellner et al. 2023) to estimate weekly detection probability (*p*) and probability of use (ψ) at each ARU station relative to activity center distance, nesting status, and barred owl calling rates (Table 1). Since the occupancy status of the eight activity center arrays was known, we treated each ARU station as a site and estimated use while accounting for imperfect detection. We used 22 weekly survey occasions and included a site‐level covariate (site_year) to account for group structure. Survey covariates on detection probability included dist, site_year, weekly barred owl detections (bo_total_wk), background noise (noise), and weekly recording effort (effort; log‐transformed; Table 1). Site covariates included dist, site_year, total seasonal barred owl detections (bo_total), and the proportion of suitable nesting‐roosting cover type within 500 m (nr_prop; Table 1). Nesting‐roosting cover type represents forest structure and tree species composition most associated with spotted owl residential use (Davis et al. 2022). We standardized (mean = 0, SD = 1) all continuous covariates unless otherwise noted. We built occupancy models sequentially, following a secondary candidate set strategy for model selection (Morin et al. 2020), and ranked models using Akaike Information Criterion (AIC).

**TABLE 1:** Descriptions and summary statistics of covariates used in single‐species, single‐season occupancy models for northern spotted owls surveyed using passive acoustic monitoring at eight activity centers.

Variable	Description	Model parameter(s)	Summary^a^
dist_*g,i*_	Actual distance between ARU and the activity center or nest tree (continuous)	ψ, *p*	Mean (SD) = 2080 m (755) Range = 0–3203 m
site_year_*g,i*_	Site identification and year (categorical)	ψ, *p*	8 activity centers
nested_*g,i*_	Indicator for whether focal owl(s) made a nest attempt (binary)	ψ, *p*	3 sites attempted to nest, 5 sites did not
bo_total_*g,i*_	Total number of predicted barred owl (BO) detections adjusted by PNW‐Cnet precision at each station across the whole season (continuous); apparent detections.	ψ	Mean (SD) = 464 (609) Range = 0–5138
bo_total_wk_*g,i,j*_	Total number of predicted barred owl (BO) detections adjusted by PNW‐Cnet precision at each station per week (continuous); apparent detections.	*p*	Mean (SD) = 25 (44) Range = 0–627
nr_prop_*g,i*_	Proportion of 30‐m^2^ cells with suitable nesting‐roosting (NR) cover (Davis et al. 2022) within a 500‐m buffer of each station (continuous)	ψ	Mean (SD) = 0.51 (0.34) Range = 0.00–1.00
noise_*g,i,j*_	Mean weekly background noise level in decibels below full scale (dBFS) (continuous)	*p*	Mean (SD) = −61.09 (7.61) Range = −70.70 to −30.10
effort_*g,i,j*_	Total number of recording minutes per station per week (logarithmic, continuous)	*p*	Mean (SD) = 2223.09 (446) Range = 19.95 to 2520.00

*Note:* Covariates were used to test for effects on detection probability (*p*) and space use (ψ) around activity center sites *g* for ARU station *i* during survey week *j*.

^a^ All continuous variables were subsequently standardized to have mean = 0 and standard deviation (SD) = 1, except effort, which was log‐transformed.

For both GLM and occupancy model analyses, we report covariate betas and 95% confidence intervals (CI), probability ratios, and created marginal effect plots. Covariate estimates with CI excluding zero were considered well supported; estimates with CI overlapping zero by ≤ 10% were considered weakly supported (MacKenzie et al. 2018).

## 3 Results

In 2021, the sites we surveyed included one non‐nesting pair, one pair with a failed nesting attempt, and two successful nests. In 2022, three of the sites we surveyed had non‐nesting pairs, and one site had a single resident female with no male detections (Table 2). Each array contained 34–37 ARUs, recording for an average of 111 days (range 6–140 days). Equipment failures, primarily due to water or wildlife damage, reduced recording duration at some stations, resulting in variation in survey effort, which we accounted for with the effort covariate in occupancy models (Table 1).

**TABLE 2:** Sample information for eight activity centers (AC) of northern spotted owls surveyed with passive acoustic monitoring.

AC	Sample period		*n*	Status	Female	Male	Any^a^
UG	4/27/2021	8/19/2021	37	N	120	1537	1621
DC	5/26/2021	9/12/2021	37	NF	37	396	438
MC	4/28/2021	9/11/2021	37	NF	10	218	228
LM	6/7/2022	9/12/2022	37	P	766	1024	1716
BC	5/3/2022	9/20/2022	37	P	543	756	1250
DC2	5/9/2022	9/21/2022	37	P	214	391	592
WC	5/5/2021	8/24/2021	34	P	171	322	429
CC	5/10/2022	9/6/2022	35	S	8	0	8

*Note:* Shown are the number of recording units (*n*) in each AC array, the reproductive status of the focal owl(s), and the number of clips containing territorial calls detected by sex and reproductive status: nesting (N), paired with no nesting (P), nesting and fledged young (NF), and single (S).

^a^ Any is the total number of clips including male, female, and unknown sex. One clip may contain both male and female calls, so Any may be more or less than the sum of male and female clips.

Across 180,001 h of audio from activity center arrays, we manually confirmed 14,114 clips containing spotted owl detections, including 6282 clips with territorial calls of any sex (Table 2). Males were more vocal than females, with 4680 male territorial calls compared to 1869 female territorial calls. Occasionally, 12‐s clips contained both male and female calls, so the total number of clips (any sex) may be less than the sum of male and female calls. On average, ARUs recorded for 19.1 weeks (SD 6.8), 38.0 (SD 5.57) hours per week, and detected 0.12 (SD 0.37) territorial calls per hour, conditional on site use.

**FIGURE 2:**
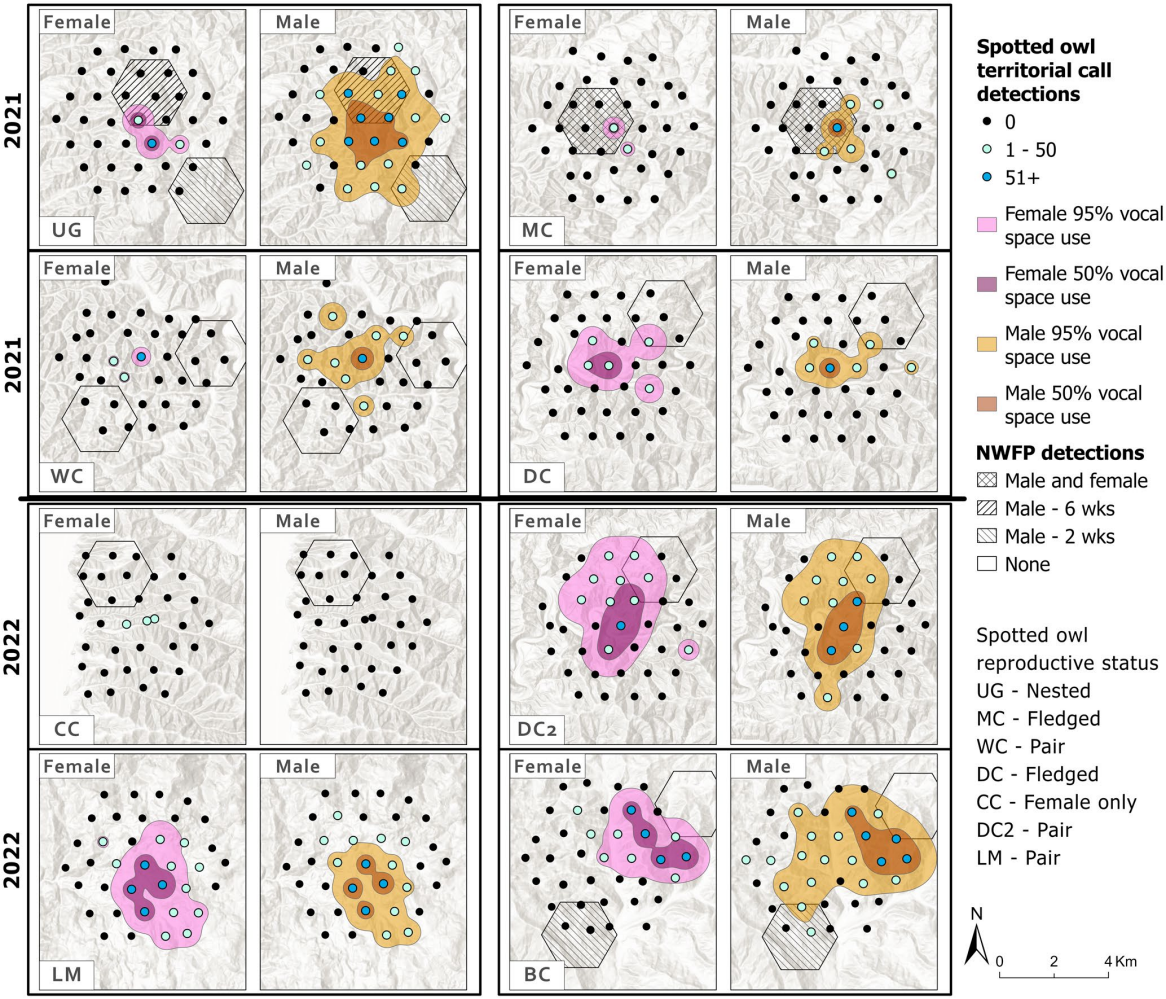
Detections of northern spotted owls from autonomous recording units (ARUs) in hexagonal arrays extending up to 3‐km radii from eight northern spotted owl activity centers, from ~22 weeks of recording. Polygons represent vocal space use areas from kernel density estimator 95% and 50% utilization distributions for vocalizations of each sex. The detection status of 5‐km^2^ Northwest Forest Plan (NWFP) monitoring hexagons sampled with four ARUs over 6–8 weeks is also shown.

Spotted owl territorial calls were detected at all activity center arrays and occurred during an average of 26% of ARU‐weeks for both female and male (SD ~23%–25%) and 30% for any sex calls (SD 27%). Spatially, most female (59.6%), male (67.6%), and any sex (64.9%) territorial calls occurred within 1 km of the activity center, with 22.1%–29.3% detected between 1 and 2 km (Figure 2). Notably, at site BC—a non‐nesting pair with low barred owl activity (19% of ARU‐weeks with barred owl detections)—the majority of spotted owl calls (> 73%) were detected beyond 2 km from the initial activity center (Figure 2). From subsequent site visits, we determined the BC pair moved their activity center to the northeast portion of the array early within our sample season; however, we did not post hoc adjust the sampling grid center. Analyses excluding site BC resulted in minor changes to covariate effects and model ranking (see Appendix B), supporting the same interpretation of covariate importance. We choose to report here the full results, including site BC, to reflect realistic noise in the detectability of spotted owls in an increasingly dynamic landscape where territorial owls may exhibit lower site fidelity than seen in decades prior (Jenkins et al. 2021). Barred owls were detected in all activity center arrays, at 277 of 290 (95.5%) recording stations, during a mean of 37% ARU‐weeks (SD 25%).

In the 10 NWFP monitoring hexagons that overlapped seven of our activity center arrays, we processed 16,817 h of audio and confirmed 485 spotted owl detections, including 252 of any sex, 51 male, and 2 female territorial calls. ARUs in these monitoring hexagons, four per hexagon, recorded for an average of 7.4 weeks (SD 1.2) at 68.2 h per week (SD 17.5), detecting 0.07 territorial calls per hour (SD 0.30). Two monitoring hexagons by chance contained two target activity centers, with male territorial calls detected across 6 and 5 weeks, respectively, and female territorial calls in the latter hexagon in a single week. Male spotted owls were also detected in two monitoring hexagons ~2 km from two target activity centers in 2 weeks (Figure 2). Barred owls were detected in nine of 10 monitoring hexagons and were vocally active in 2–7 survey weeks.

### 3.1 Vocal Space Use

**FIGURE A1:**
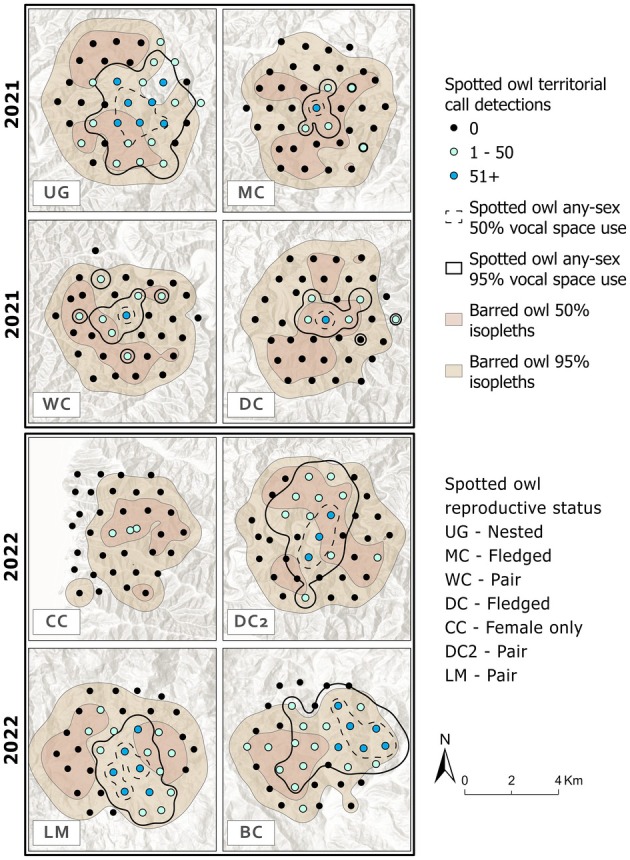
Overlap between northern spotted owl and barred owl kernel density estimator 50% and 95% utilization distributions (UDs) surrounding eight active spotted owl activity centers between April and September 2021 and 2022. Spotted owl UDs reflect individual (CC) or pair vocal space use. Barred owl UDs reflect study array coverage of barred owl vocal activity.

Kernel density estimates showed variation in vocal space use by site and sex (Figure 2, Table A3). At site CC, a single female was detected only three times, insufficient for generating UDs. Elsewhere, sample sizes for females at two sites were potentially below recommended thresholds (< 30 detections—Seaman et al. 1999; < 15 detections—Anich et al. 2009; Table 2), though we still calculated UDs for demonstration because female detections are rare. Mean 50% vocal space use areas were similar for females (143.9 ha; 179.4 ha excluding low‐sample sites), males (192.5 ha), and any sex (191.6 ha). Mean 95% vocal space use areas differed for female (647.2 ha; 760.7 ha excluding small sample sites), male (1025.2 ha), and any sex (998.0 ha). Barred owl vocalization coverage was high, with generalized vocal activity 50% isopleths ranging from 682 to 1262 ha (16.4%–30.4% coverage) and 95% isopleths from 2108 to 3559 ha (50.7%–85.6% coverage; Figure A1).

### 3.2 Generalized Linear Models: Patterns of Vocal Activity

**FIGURE 3:**
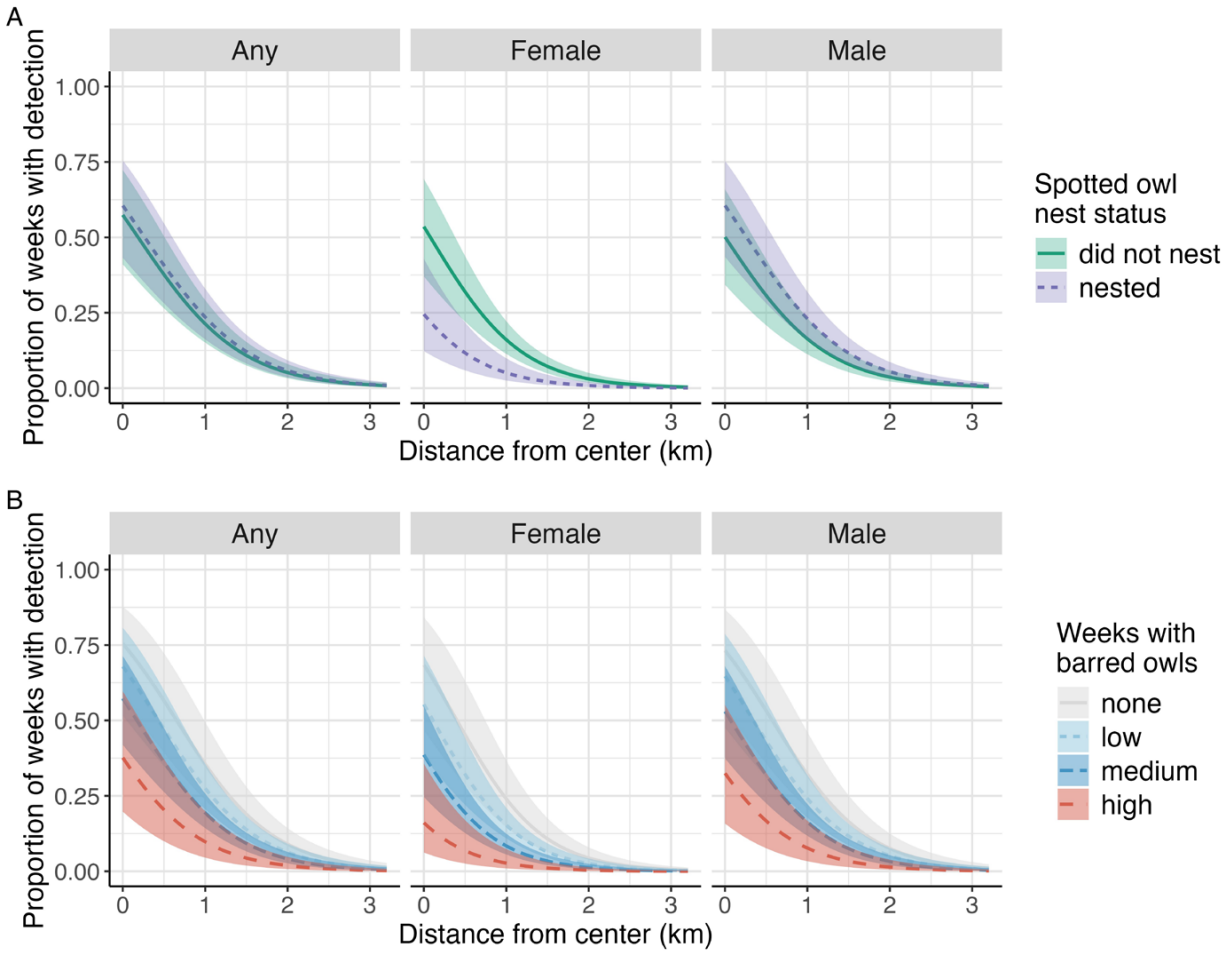
Marginal plots generated from generalized linear models relating the proportion of weeks with detection of northern spotted owl territorial calls by sex and combined (any sex calls) by distance from activity center. Panel A: Additive effect of nesting status; Panel B: Additive effect of magnitude of barred owl calling measured in proportion of weeks with barred owl detections (low < 0.192, medium = 0.192–0.471, high > 0.471).

**FIGURE B1:**
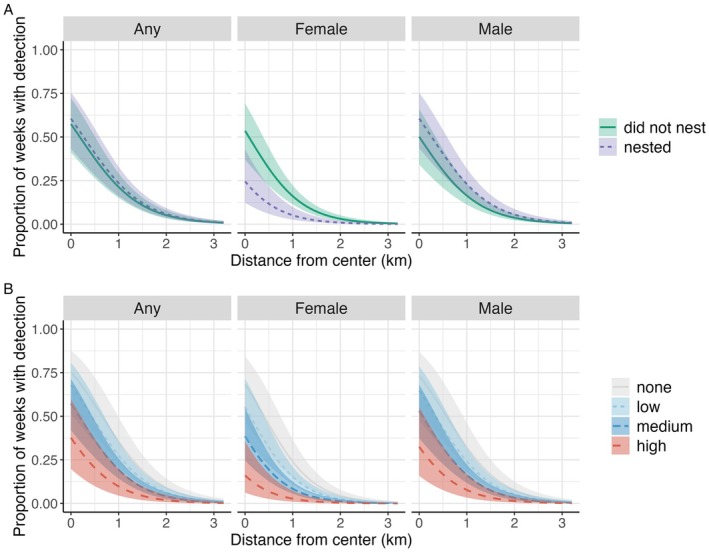
Marginal plots generated from generalized linear models, excluding site BC, relating the proportion of weeks with detection of northern spotted owl territorial calls by sex and combined (any sex calls) by distance from activity center. Panel A: Additive effect of nesting status; Panel B: Additive effect of magnitude of barred owl calling measured in proportion of weeks with barred owl detections (low < 0.192, medium = 0.192–0.471, high > 0.471).

Calling rate (i.e., proportion of weeks with detection) consistently declined with distance from the activity center across all datasets (female, male, any sex; Figure 3; Figure B1) and models (Table 3; Table B1). The proportion of weeks with any spotted owl territorial calls decreased from 0.56 (CI: 0.39–0.72) at the activity center to 0.24 (CI: 0.16–0.34) 1 km from the activity center, an approximate 57% decrease (Figure 3). Nesting status influenced female territorial calling only, with nesting females calling less frequently (0.21 of weeks, CI: 0.10–0.39) than non‐nesting females (0.43 of weeks, CI: 0.29–0.58), an approximate 51% decline (Figure 3).

**TABLE 3:** Coefficient estimates (β) with standard errors (SE) and *p*‐values from quasibinomial generalized linear models (GLMs) for northern spotted owls, using the proportion of weeks with spotted owl detections by sex (any = all territorial calls regardless of sex) as the response variable.

Model	Coefficient	Any		Female		Male	
		β (SE)	*p*	β (SE)	*p*	β (SE)	*p*
~dist	(Intercept)	0.028 (0.297)	0.92	−0.742 (0.349)	0.03	−0.121 (0.289)	0.67
	dist	−0.001 (0.0002)	< 0.01	−0.001 (0.0002)	< 0.01	−0.001 (0.0002)	< 0.01
~dist + nested	(Intercept)	0.049 (0.313)	0.88	−0.390 (0.330)	0.24	−0.185 (0.311)	0.55
	dist	−0.001 (0.0002)	< 0.01	−0.001 (0.0002)	< 0.01	−0.001 (0.0002)	< 0.01
	nested(y)	−0.059 (0.292)	0.84	−1.225 (0.434)	< 0.01	0.176 (0.291)	0.55
~dist + bo_total	(Intercept)	0.454 (0.353)	0.20	−0.021 (0.414)	0.96	0.285 (0.347)	0.41
	dist	−0.001 (0.0002)	< 0.01	−0.001 (0.0002)	< 0.01	−0.001 (0.0002)	< 0.01
	bo_total	−0.001 (0.0002)	0.05	−0.001 (0.0004)	0.02	−0.0004 (0.0002)	0.06
~dist + bo_prop	(Intercept)	0.735 (0.381)	0.05	0.288 (0.426)	0.50	0.599 (0.380)	0.12
	dist	−0.001 (0.0002)	< 0.01	−0.001 (0.0002)	< 0.01	−0.001 (0.0002)	< 0.01
	bo_prop	−1.596 (0.575)	< 0.01	−2.449 (0.737)	< 0.01	−1.633 (0.587)	< 0.01

*Note:* See Table 1 for covariate definitions and Figure 3 to visualize key effects.

Recurring barred owl calling (bo_prop) was associated with lower calling rates in all datasets, while total counts of barred owl calls (bo_total) had a weaker effect (Table 3). We found a stronger effect of recurring barred owl calling (bo_prop) on female calls (β = −2.4; CI: −3.9 to −1.0) than the effect on any sex calls (β = −1.6; CI: −2.7 to −0.5) and male calls (β = −1.6; CI: −2.8 to −0.5). The total counts of barred owl calls (bo_total) had a weaker effect across all datasets (β = −0.001; CI: −0.0006 to −0.0013). The proportion of weeks with any sex spotted owl territorial calls declined by approximately 54% when the proportion of weeks with barred owl detections increased from 0.1 to 0.6, at the mean distance value, from 0.08 (CI: 0.06–0.12) to 0.04 (CI: 0.02–0.07), respectively (Figure 3). The proportion of weeks with female spotted owl calls declined by approximately 65% when the proportion of weeks with barred owl detections increased from 0.1 to 0.6, at the mean distance value, from 0.05 (CI: 0.03–0.08) to 0.02 (CI: 0.01–0.04), respectively (Figure 3).

### 3.3 Occupancy Models: Detection Probability and Site Use

**FIGURE 4:**
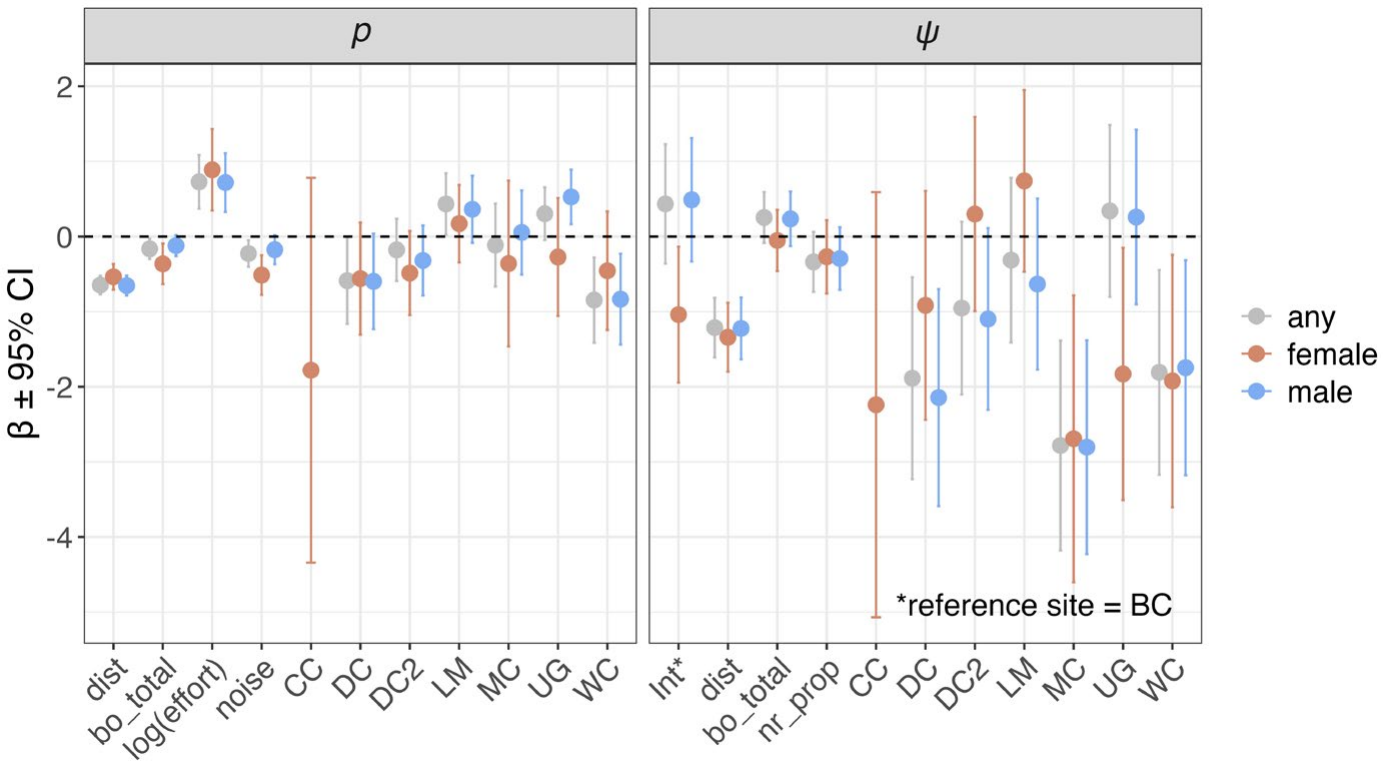
Covariate beta coefficients (β) and 95% confidence intervals (CI) for northern spotted owl probability of use (ψ) and detection probability (*p*) from data collected using passive acoustic monitoring around eight activity centers over ~22 weeks in 2021 and 2022. The illustrated model was the highest‐ranked for any sex spotted owl calls and within 3 Akaike's Information Criterion of the most‐supported model for female and male calls. See Table 1 for covariate definitions and Table A2 for *p*(Int) and exact values.

**FIGURE 5:**
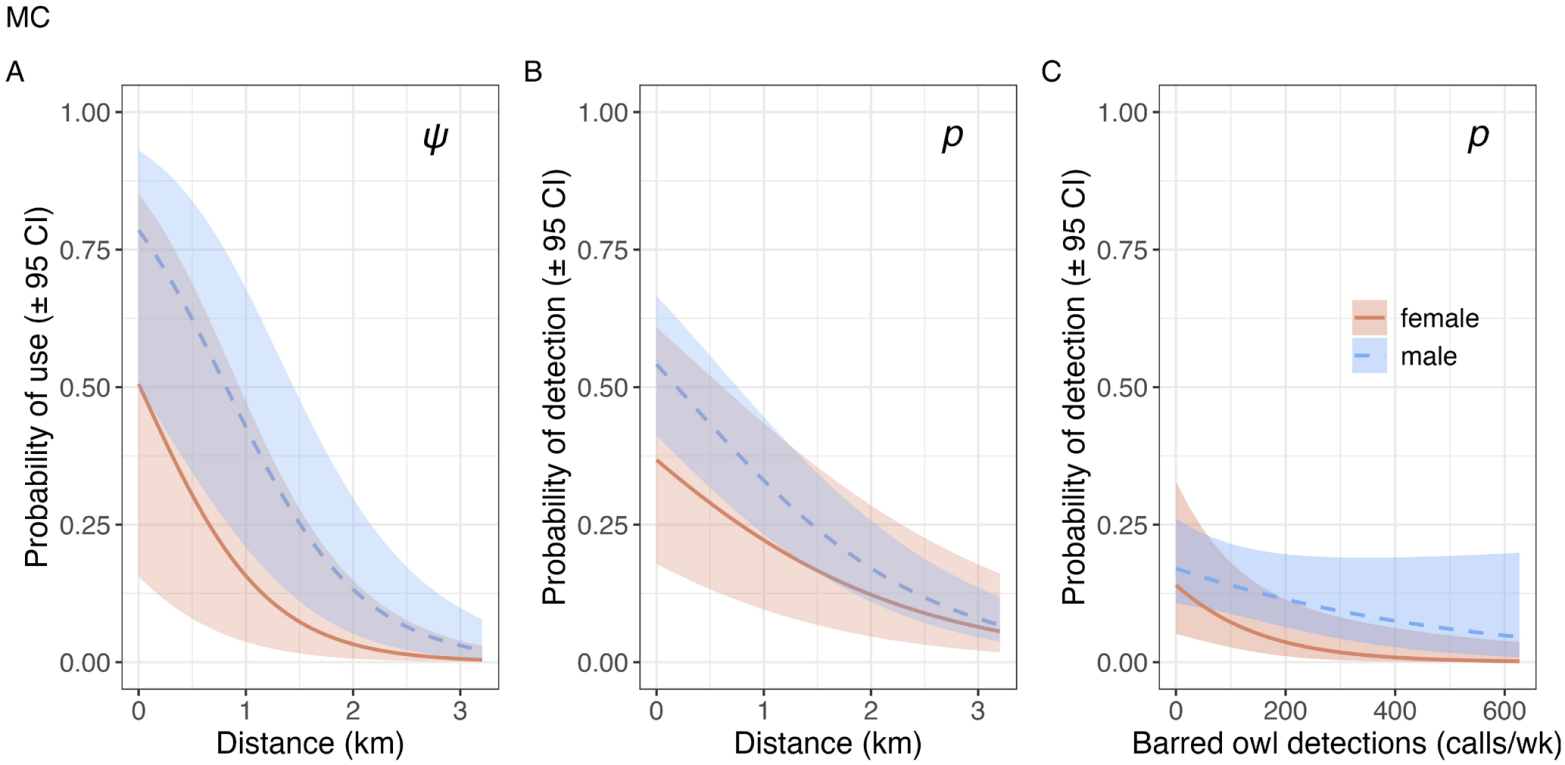
Marginal plots (95% confidence intervals) for the effect of distance (dist) on the probability of use (ψ; panel A) and effects of distance (dist) and barred owl detections per week (bo_total_wk) on detection probability (*p*; panels B and C, respectively) for female and male northern spotted owls, generated from the most‐supported occupancy models. The most‐supported model also included effects of noise, effort, and site_year (see Figure A2). Plots shown are for site MC, where continuous non‐focal covariates were held at their means.

**FIGURE A2:**
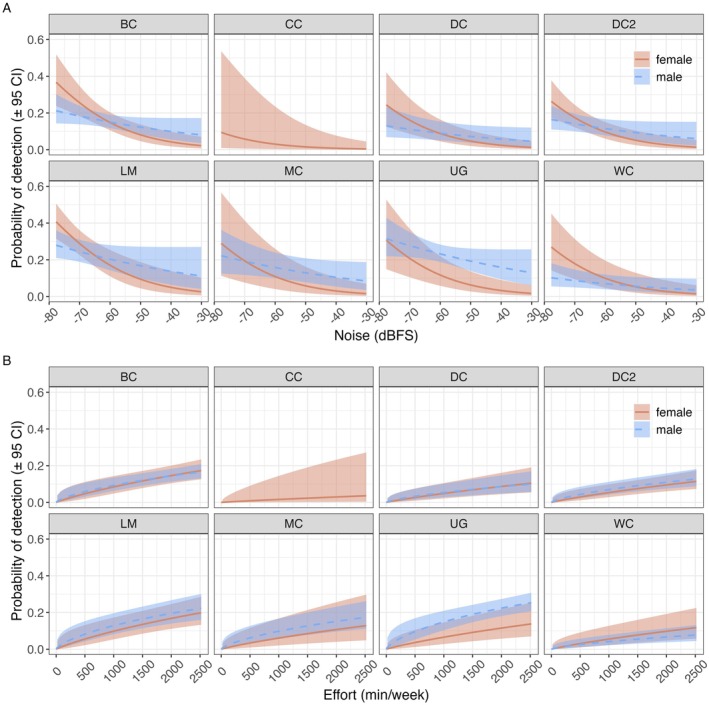
Marginal plots (95% confidence intervals) for female and male northern spotted owls from the most‐supported occupancy model, which included effects of distance to activity center (Figure 5), activity center site, background noise measured in dBFS (decibels below full scale; panel A), survey effort (panel B), and total barred owl calls per week (Figure 5) on detection probability (*p*). Effects on probability of use (ψ) included distance to activity center (Figure 5) and activity center site. Plots shown hold continuous non‐focal covariates at their mean. See main text Table 1 for covariate definitions.

**FIGURE B2:**
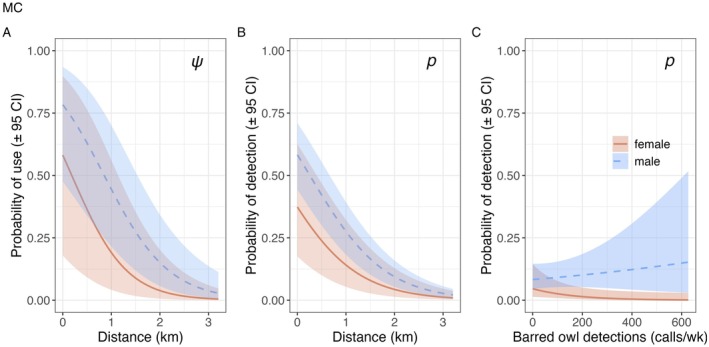
Marginal plots (95% confidence intervals) for the effect of distance (dist) on the probability of use (ψ; panel A) and the effects of distance (dist) and barred owl detections per week (bo_total_wk) on detection probability (*p*; panels B and C, respectively) for female and male northern spotted owls, generated from the most‐supported occupancy model, excluding site BC. The most‐supported model also included the effects of noise, effort, and site_year. Plots are shown for site MC, and continuous non‐focal covariates were held at their mean.

The most‐supported detection probability model for female, male, and any sex calls included site_year, the negative effect of increasing noise, dist, bo_total, and a positive effect of increasing effort (Table 4; Figure 4; Tables A1 and A2; Figures A2 and B2). The effect of bo_total was slightly negative with weak support for male calls (β = −0.10; CI: −0.23 to 0.02) and any sex calls (β = −0.14; CI: −0.25 to 0.03), while the negative effect on female calls was supported (β = −0.33; CI: −0.56 to −0.09; Figure 4). Weekly detection probability decreased by 40% for females from 0.37 (CI: 0.18–0.61) at the activity center to 0.22 (CI: 0.10–0.43) at 1 km from the activity center and by 51% for males from 0.54 (CI: 0.41–0.67) at the activity center to 0.33 (CI: 0.23–0.45) at 1 km from the activity center (Figure 5).

**TABLE 4:** Most‐supported occupancy models (> 0.90 cumulative model weight) ranked by Akaike's Information Criterion (AIC) for northern spotted owls, including number of parameters (K), model weight (*w*), and model structure for probability of detection (*p*) and of use (ψ).

Sex	Model structure	K	ΔAIC	AIC	*w*
Female	*p*~dist + site_year + noise + effort + bo_total_wk ψ~dist + site_year	21	0	1135.1	0.58
	*p*~dist + site_year + noise + effort + bo_total_wk ψ~dist + site_year + bo_total	22	1.90	1137.0	0.22
	*p*~dist + site_year + noise + effort + bo_total_wk ψ~dist + site_year + bo_total + nr_prop	23	2.80	1137.9	0.13
Male	*p*~dist + site_year + noise + effort + bo_total_wk ψ~dist + site_year	21	0	1838.2	0.42
	*p*~dist + site_year + noise + effort + bo_total_wk ψ~dist + site_year + bo_total	22	0.69	1838.9	0.30
	*p*~dist + site_year + noise + effort + bo_total_wk ψ~dist + site_year + bo_total + nr_prop	23	0.76	1838.9	0.30
Any	*p*~dist + site_year + noise + effort + bo_total_wk ψ~dist + site_year + bo_total + nr_prop	23	0	2009.0	0.43
	*p*~dist + site_year + noise + effort + bo_total_wk ψ~dist + site_year	21	0.75	2009.7	0.29
	*p*~dist + site_year + noise + effort + bo_total_wk ψ~dist + site_year + bo_total	22	0.83	2009.8	0.28

*Note:* See Table 1 for covariate definitions and Table A1 for the full model set.

The most‐supported models of ψ included dist and site_year for female and male spotted owls (Table 4; Figure 4; Tables B2 and B3; Figure B2). For any sex, the highest‐ranked model also included bo_total and nr_prop, though the CI overlapped zero, indicating no support (Table A2). Probability of use at ARU stations decreased by 69% for females from 0.51 (CI: 0.16–0.85) at the activity center to 0.16 (CI: 0.04–0.47) at 1 km from the activity center and by 46% for males from 0.79 (CI: 0.50–0.93) at the activity center to 0.43 (CI: 0.21–0.68) at 1 km from the activity center (Figure 5).

## 4 Discussion

Monitoring space use by cryptic, territorial species presents a universal challenge in ecology and conservation (Ross et al. 2023; Sugai et al. 2018). Passive acoustic monitoring has emerged as a powerful tool for assessing occupancy and behavior at scale, yet interpreting detections requires understanding species‐specific calling patterns, space use, and the influence of ecological context (Lesmeister and Jenkins 2022; Tosa et al. 2021). Our study of northern spotted owls demonstrates one approach to address this challenge and provides a resource to refine occupancy inference from randomized monitoring programs, offering broader lessons for monitoring territorial species in dynamic landscapes.

We show that spotted owl vocal activity is tightly centered around core use areas, with detection probability and calling rates declining sharply with distance from activity centers, especially for females. Males were more frequently detected than females, and male call patterns did not vary much with nesting status, while females were rarely detected ≥ 1 km from an active nest. These patterns reflect fundamental aspects of territorial behavior, especially for central place foragers—vocalizations mark defended space but do not encompass the full extent of home range use (Chronister et al. 2025; Ophir et al. 2010; Reid et al. 2022). For example, Hamer et al. (2007) suggested that home ranges encompass foraging areas too large to defend effectively. Across our study, 50% vocal space use areas averaged 144–193 ha, approximating previous core use estimates (87–100 ha; Glenn et al. 2004), and our 95% areas (647–1025 ha) may reflect vocalizations across much (but not all) of estimated full home ranges (422–3608 ha; Hamer et al. 2007). This spatially structured variation in detectability has important implications for interpreting acoustic data in occupancy models or for timber project clearance, especially when activity center locations are unknown.

For passive acoustic monitoring, our data suggests an ARU that detects frequent territorial calling has a listening radius that likely overlaps an activity center, while infrequent detections may be indicative of sampling within the home range farther from core areas. Such patterns extend beyond our focal system; territorial species globally rely on vocal or acoustic signals to defend space, attract mates, and mediate competition (Ophir et al. 2010). Acoustic detection probabilities are thus not only species‐ and sex‐specific but also deeply entwined with ecological context, including reproductive status, competitor presence, and habitat structure (Chronister et al. 2025). Our results illustrate how ecological pressures, like competition from a non‐native congener, can further shape calling behavior and detection outcomes (Appel et al. 2023; Rugg et al. 2023; Wiens et al. 2014).

In GLM analysis, recurring barred owl calling was negatively associated with overall spotted owl calling relative to sites with low rates or no barred owl calling, particularly for females whose calls were already less frequent. While barred owls were detected at most ARU stations, higher calling rates may reflect higher population densities of barred owls (Wiens et al. 2021), resulting in greater competition. The risk of predation or competition from either barred owls or great‐horned owls (Paton et al. 1991; Wiens et al. 2021) and the energetic costs of vocalizations (Ophir et al. 2010) limit the areas spotted owls can effectively defend. These findings align with other studies showing reduced vocal activity and detectability of native species in the presence of dominant competitors (Duchac et al. 2020; Rugg et al. 2023; Wiens et al. 2014, 2021). Suppressed calling may have direct consequences for occupancy inference, increasing the likelihood of false negatives in areas with intense competition.

Detection thresholds (i.e., a minimum number of detections to establish occupancy) offer a simple approach to reducing error in interpreting acoustic results in ecological studies (Reid et al. 2021), but choosing an appropriate threshold depends on ecological context, study objective, and risk tolerance. For population monitoring that generates landscape‐scale estimates, stricter criteria—such as detections across multiple weeks and of both sexes—reduce false positives in determining pair occupancy (Berigan et al. 2019). However, when the goal is habitat protection, especially under precautionary management, more inclusive thresholds can reduce false negatives and help identify greater portions of the home range. Practitioners may utilize multiple thresholds, allowing for identification of both likely core use areas and additional landscapes used by territorial owls. We surveyed one territory with a single female spotted owl, detected only three times in 18 survey weeks at the activity center. This underscores that even isolated detections, particularly of females, may signal latent territory occupancy or recruitment potential, especially in landscapes with low densities and shifting population dynamics (Davis et al. 2022; Franklin et al. 2021; Jenkins et al. 2021).

**FIGURE 6:**
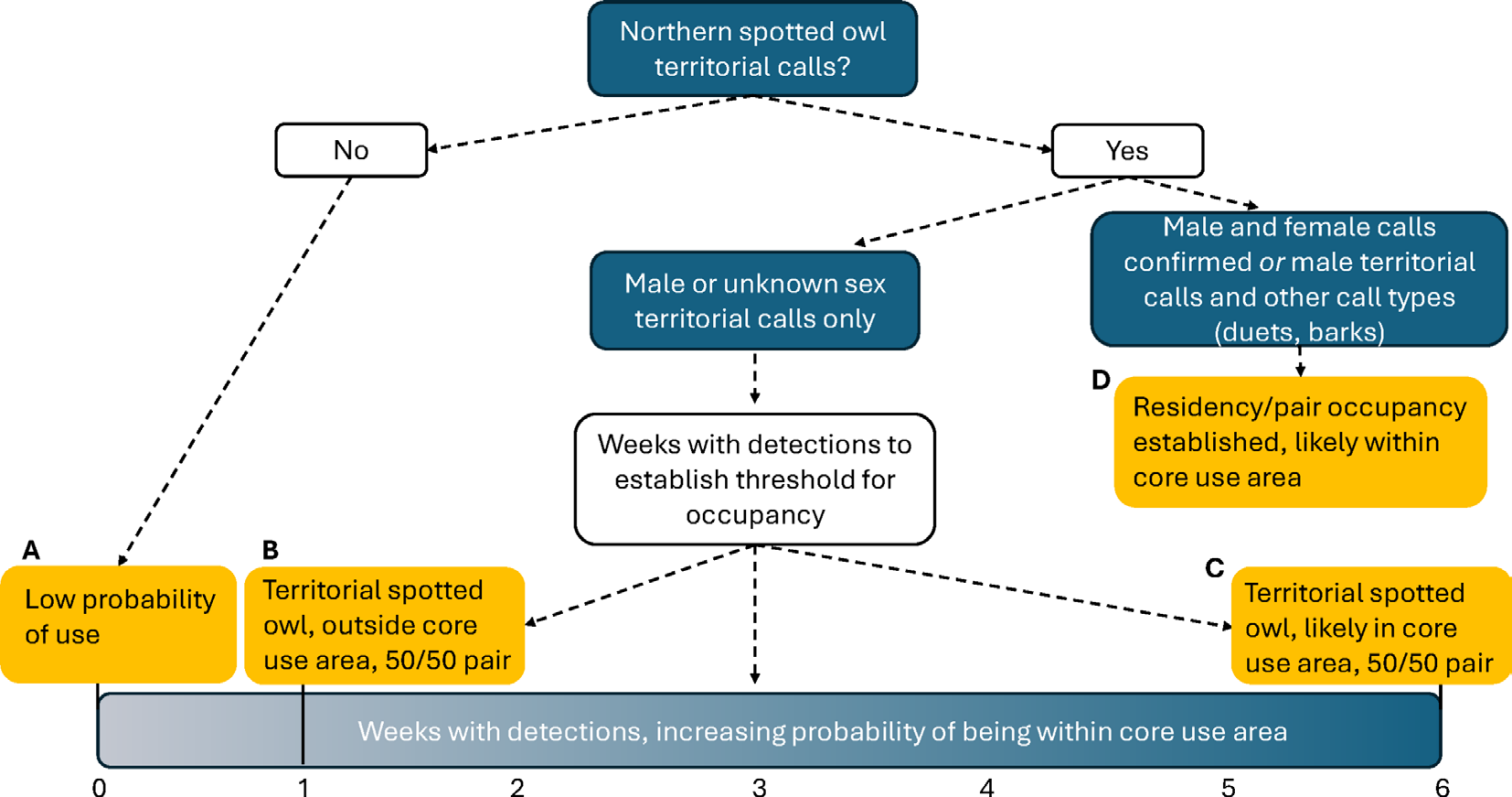
Spectrum for interpreting northern spotted owl territorial call detections from passive acoustic monitoring surveys: Low probability of use with no territorial call detections (A); when detected for 1 or 2 weeks, detected outside of core use area (B); detected for 4–6 weeks, detected within the core use area (C). In both cases of B and C, when only a male is detected, there is an approximate 50% probability of being paired, but the female was undetected (Appel et al. 2023). Detection of both sexes confirms pair occupancy with a high likelihood that the survey was within the core use area (D).

We propose a flexible, detection‐based framework that incorporates calling rates, caller sex, and survey objectives to guide interpretation of acoustic survey data for northern spotted owls in the breeding season (Figure 6). This framework is especially useful when locations of activity centers or active territories are unknown. Because these calls are territorial in nature, their unelicited detection reliably indicates use by resident owls rather than non‐residents. The key uncertainty is not whether the owl is territorial, but whether the detection occurred within the owl's core use area or in a peripheral area of its home range. High calling rates or detections of both sexes strongly suggest an overlap with the core use area, while intermediate detection patterns indicate proximity to core areas. Sparse detections likely reflect use within the broader home range and greater distance from the activity center, potentially reflecting foraging areas. This framework supports adaptive management, allowing practitioners to calibrate thresholds based on risk tolerance and conservation priorities.

These recommendations are based on the standard survey design used by the NWFP effectiveness monitoring program—four ARUs deployed per 5‐km^2^ hexagon and recording ~10 h per day for 6 weeks (Lesmeister et al. 2021). Surveys with reduced effort may be less likely to detect natural calling behavior and should not be interpreted using the same thresholds. Ultimately, the appropriate threshold for interpreting detection data depends on project goals, whether maximizing confidence in occupancy for demographic modeling or ensuring sufficient habitat protection for conservation planning. For example, passive acoustic monitoring at the 5‐km^2^ hexagon scale—the regional NWFP population monitoring design (Lesmeister and Jenkins 2022)—successfully identified territorial spotted owls at four sites, without prior knowledge of activity centers. Two hexagons overlapping activity centers of pairs detected males in at least 5 weeks, and one detected both sexes, providing strong evidence of true occupancy. However, detections in monitoring hexagons not overlapping known activity centers captured male territorial calls in 2 weeks—indicative of peripheral space use. Under strict thresholds, researchers could effectively determine occupancy in the former two hexagons as well as likely core use area locations and broader landscape use in the latter, reducing uncertainty in the observed biological status of the site.

Importantly, surveys are imperfect, so the absence of detections did not equate to the absence of owls (MacKenzie et al. 2018). Several NWFP hexagons overlapped estimated vocal space use areas but recorded no detections, reinforcing that passive acoustic monitoring is subject to imperfect detection. While territorial defense likely indicates high‐use areas, ARUs in key foraging areas of the home range may not detect territorial vocalizations. This caution is virtually universal (MacKenzie et al. 2018): silence does not guarantee absence, particularly when calling rates are low, environmental noise is high, or when animals are intentionally cryptic while foraging. However, passive acoustic monitoring benefits from high survey effort with little human effort, resulting in higher seasonal detection probabilities for rare and elusive species than active survey methods (Duchac et al. 2020). Incorporating detection probability estimates into spotted owl occupancy models is essential for robust inference and minimizes the risk of non‐detection at an occupied site (Appel et al. 2023; Duchac et al. 2020).

Studies on behavioral ecology provide critical context for interpreting detection patterns (Chronister et al. 2025), while occupancy models help quantify uncertainty and guide decision‐making. In dynamic landscapes, where species interactions and habitat conditions are shifting (Davis et al. 2022; Jenkins et al. 2021), this integrated approach enables more accurate and actionable monitoring. For the northern spotted owl, such refinement is urgently needed. Despite a slower rate of old‐growth forest loss on federally administered forest lands, populations continue to decline, driven by habitat loss and barred owl competition (Franklin et al. 2021). Passive acoustic monitoring offers a scalable, cost‐effective tool for tracking population trends and informing management, but its effectiveness depends on clear interpretation frameworks (Lesmeister et al. 2021). Future refinements to our approach could include expanding call types analyzed beyond the territorial defense calls. For example, female spotted owls use territorial calls less frequently than males, and other call types, such as barks, may be reliable indicators of sex and behavioral context (Forsman et al. 1984; Dale et al. 2022). Incorporating these alternative calls into encounter histories could improve detection probability of females and refine occupancy estimates (Reid et al. 2022). However, caution is needed to avoid misclassification, especially where call types overlap with those of barred owls and other species (Odom and Mennill 2010; Forsman et al. 1984).

Looking beyond the Pacific Northwest, our approach offers a template for monitoring other territorial cryptic species, especially where vocalizations provide reliable indicators of presence and behavior (Chronister et al. 2025). Whether applied to rainforest primates, territorial songbirds, or bats, integrating calling rate, spatial context, and ecological understanding enhances the power of passive acoustic monitoring for conservation. Ultimately, effective conservation requires tools that are both scientifically robust and practically applicable. By clarifying how vocal behavior reflects space use and occupancy, this study advances passive acoustic monitoring as a reliable method for detecting, understanding, and conserving territorial species in complex landscapes. For the northern spotted owl, this means more accurate identification of occupied sites, better‐informed habitat protection, and stronger foundations for recovery amid the dual pressures of habitat change and novel competitors.

## Author Contributions

**Natalie M. Rugg:** data curation (equal), visualization (supporting), writing – original draft (lead). **Cara L. Appel:** data curation (equal), formal analysis (lead), investigation (equal), methodology (equal), software (equal), visualization (lead), writing – review and editing (equal). **Julianna M. A. Jenkins:** data curation (equal), formal analysis (supporting), investigation (equal), methodology (equal), software (equal), supervision (equal), visualization (supporting), writing – review and editing (equal). **Chris McCafferty:** conceptualization (equal), investigation (equal), writing – review and editing (equal). **Taal Levi:** conceptualization (equal), methodology (equal), supervision (equal), validation (equal), writing – review and editing (equal). **Damon B. Lesmeister:** conceptualization (equal), funding acquisition (lead), methodology (equal), project administration (lead), resources (lead), supervision (equal), validation (equal), writing – review and editing (equal).

## Conflicts of Interest

The authors declare no conflicts of interest.

## Data Availability

Rugg, N., Appel, C., Jenkins, J., McCafferty, C., Levi, T., and Lesmeister, D. (2025). Detection histories and covariates for northern spotted owl vocal space use [Data set]. *Zenodo*. https://doi.org/10.5281/zenodo.15346260.

## Appendix A

**TABLE A1:** Full suite of single‐species single‐season occupancy models ranked by difference in Akaike's Information Criterion (AIC) for northern spotted owls, including number of parameters (K), Akaike's model weight (*w*), cumulative Akaike's model weight (cumwt), and model structure for detection probability (*p*) and probability of use (ψ).

Sex	Model structure	K	AIC	ΔAIC	*w*	Cumwt
Female	*p~*dist + site_year + noise + log(effort) + bo_total_wk ψ~dist + site_year	21	1135.1	0.0	0.58	0.58
	*p~*dist + site_year + noise + log(effort) + bo_total_wk ψ~dist + site_year + bo_total	22	1137.0	1.9	0.22	0.79
	*p~*dist + site_year + noise + log(effort) + bo_total_wk ψ~dist + site_year + bo_total + nr_prop	23	1137.9	2.7	0.13	0.92
	*p~*dist + site_year + noise + log(effort) + bo_total_wk ψ~dist	14	1150.8	15.7	0.05	0.97
	*p~*dist + site_year + noise + log(effort) ψ~dist	13	1156.0	20.9	0.03	1
	*p~*dist + site_year + log(effort) ψ~dist	12	1168.5	33.4	0	1
	*p~*dist + site_year + noise ψ~dist	12	1168.8	33.6	0	1
	*p~*dist + site_year ψ~dist	11	1184.4	49.3	0	1
	*p~*dist + nest ψ~dist	5	1224.6	89.5	0	1
	*p~*dist ψ~dist	4	1229.7	94.5	0	1
	*p~*1 ψ~1	2	1287.3	152.1	0	1
Male	*p~*dist + site_year + noise + log(effort) + bo_total_wk ψ~dist + site_year	21	1838.2	0.0	0.42	0.42
	*p~*dist + site_year + noise + log(effort) + bo_total_wk ψ~dist + site_year + bo_total	22	1838.9	0.7	0.30	0.71
	*p~*dist + site_year + noise + log(effort) + bo_total_wk ψ~dist + site_year + bo_total + nr_prop	23	1838.9	0.8	0.29	1
	*p~*dist + site_year + noise + log(effort) + bo_total_wk ψ~dist	14	1851.8	13.7	0	1
	*p~*dist + site_year + noise + log(effort) ψ~dist	13	1852.2	14.1	0	1
	*p~*dist + site_year + log(effort) ψ~dist	12	1853.6	15.4	0	1
	*p~*dist + site_year + noise ψ~dist	12	1868.9	30.8	0	1
	*p~*dist + site_year ψ~dist	11	1871.7	33.5	0	1
	*p~*dist + nest ψ~dist	5	1940.4	102.3	0	1
	*p~*dist ψ~dist	4	1941.3	103.1	0	1
	*p~*1 ψ~1	2	2073.9	235.7	0	1
Any	*p~*dist + site_year + noise + log(effort) + bo_total_wk ψ~dist + site_year + bo_total + nr_prop	23	2009.0	0.0	0.43	0.43
	*p~*dist + site_year + noise + log(effort) + bo_total_wk ψ~dist + site_year	21	2009.7	0.7	0.29	0.72
	*p~*dist + site_year + noise + log(effort) + bo_total_wk ψ~dist + site_year + bo_total	22	2009.8	0.8	0.28	1
	*p~*dist + site_year + noise + log(effort)+bo_total_wk ψ~dist	14	2024.7	15.8	0	1
	*p~*dist + site_year + noise + log(effort) ψ~dist	13	2027.4	18.5	0	1
	*p~*dist + site_year + log(effort) ψ~dist	12	2031.2	22.2	0	1
	*p~*dist + site_year + noise ψ~dist	12	2047.1	38.1	0	1
	*p~*dist + site_year ψ~dist	11	2052.9	43.9	0	1
	*p~*dist ψ~dist	4	2142.4	133.4	0	1
	*p~*dist + nest ψ~dist	5	2144.3	135.4	0	1
	*p~*1 ψ~1	2	2272.1	263.2	0	1

*Note:* Models used detections of female, male, or any sex spotted owl territorial calls collected at eight activity centers. See Table 1 in the main text for covariate definitions.

**TABLE A2:** Covariate beta coefficients (β), lower 95% confidence limit (LCL), and upper 95% confidence limit (UCL) reflecting effects on probability of use (ψ) and detection probability (*p*) for northern spotted owls using detections of female, male, or any sex of territorial calls collected at eight activity centers.

Group	Parameter	Covariate	β	LCL	UCL
Female	ψ	Intercept	−1.04	−1.94	−0.13
	ψ	dist	−1.34	−1.80	−0.88
	ψ	site_yearCC	−2.24	−5.07	0.60
	ψ	site_yearDC	−0.90	−2.43	0.62
	ψ	site_yearDC2	0.30	−0.99	1.59
	ψ	site_yearLM	0.74	−0.47	1.95
	ψ	site_yearMC	−2.71	−4.61	−0.81
	ψ	site_yearUG	−1.83	−3.51	−0.15
	ψ	site_yearWC	−1.92	−3.60	−0.24
	ψ	bo_total	−0.05	−0.46	0.36
	ψ	nr_prop	−0.27	−0.76	0.22
	*p*	Intercept	−12.16	−18.61	−5.70
	*p*	dist	−0.54	−0.71	−0.37
	*p*	site_yearCC	−1.79	−4.35	0.78
	*p*	site_yearDC	−0.59	−1.33	0.16
	*p*	site_yearDC2	−0.49	−1.05	0.07
	*p*	site_yearLM	0.17	−0.35	0.69
	*p*	site_yearMC	−0.36	−1.46	0.74
	*p*	site_yearUG	−0.27	−1.06	0.51
	*p*	site_yearWC	−0.46	−1.25	0.33
	*p*	Noise	−0.46	−0.70	−0.23
	*p*	log(effort)	0.89	0.35	1.43
	*p*	bo_total_wk	−0.32	−0.56	−0.08
Male	ψ	Intercept	0.49	−0.33	1.31
	ψ	dist	−1.23	−1.64	−0.82
	ψ	site_yearCC	−10.89	NA	NA
	ψ	site_yearDC	−2.15	−3.59	−0.70
	ψ	site_yearDC2	−1.10	−2.31	0.11
	ψ	site_yearLM	−0.63	−1.77	0.51
	ψ	site_yearMC	−2.82	−4.24	−1.40
	ψ	site_yearUG	0.25	−0.91	1.41
	ψ	site_yearWC	−1.76	−3.19	−0.33
	ψ	bo_total	0.24	−0.13	0.60
	ψ	nr_prop	−0.30	−0.71	0.12
	*p*	Intercept	−10.17	−14.80	−5.55
	*p*	dist	−0.65	−0.78	−0.52
	*p*	site_yearCC	−8.51	NA	NA
	*p*	site_yearDC	−0.60	−1.23	0.04

	*p*	site_yearDC2	−0.32	−0.78	0.15
	*p*	site_yearLM	0.36	−0.08	0.81
	*p*	site_yearMC	0.06	−0.50	0.62
	*p*	site_yearUG	0.53	0.17	0.89
	*p*	site_yearWC	−0.83	−1.43	−0.23
	*p*	noise	−0.16	−0.33	0.02
	*p*	log(effort)	0.72	0.33	1.11
*p*	bo_total_wk	−0.11	−0.23	0.02
Any	ψ	Intercept	0.45	−0.34	1.24
	ψ	dist	−1.16	−1.54	−0.78
	ψ	site_yearCC	−1.80	−6.38	2.78
	ψ	site_yearDC	−1.75	−3.06	−0.43
	ψ	site_yearDC2	−0.87	−2.00	0.27
	ψ	site_yearLM	−0.38	−1.47	0.70
	ψ	site_yearMC	−2.44	−3.75	−1.12
	ψ	site_yearUG	0.15	−0.97	1.27
	ψ	site_yearWC	−1.69	−2.97	−0.41
	ψ	bo_total	0.25	−0.09	0.60
	ψ	nr_prop	−0.14	−0.52	0.24
	*p*	Intercept	−10.69	−14.86	−6.53
	*p*	dist	−0.68	−0.79	−0.56
	*p*	site_yearCC	−2.99	−5.40	−0.57
	*p*	site_yearDC	−0.60	−1.14	−0.06
	*p*	site_yearDC2	−0.20	−0.59	0.20
	*p*	site_yearLM	0.58	0.18	0.98
	*p*	site_yearMC	0.17	−0.34	0.67
	*p*	site_yearUG	0.35	0.00	0.69
	*p*	site_yearWC	−0.59	−1.11	−0.07
	*p*	Noise	−0.31	−0.46	−0.16
	*p*	log(effort)	0.78	0.43	1.13
	*p*	bo_total_wk	−0.14	−0.25	−0.03

*Note:* The model shown had the most support for any sex northern spotted owl calls and was within 3 ΔAIC of the top‐ranked model for female and male spotted owls, which did not include an effect of barred owl activity or nesting‐roosting forest on ψ. See main text Table 1 for covariate definitions and Figures 4 and 5 for visualization of effects.

**TABLE A3:** Kernel density estimator 50% and 95% utilization distribution (UD) isopleths (measured in hectares) calculated using days with detections of male, female, and any sex spotted owl territorial call detections from eight activity centers.

Site	Any 95%	Any 50%	Female 95%	Female 50%	Male 95%	Male 50%	Sample: any	Sample: female	Sample: male
DC	446.8	48.6	654.0	98.5	396.4	43.7	64	20^a^	49
MC	288.9	34.0	73.1	11.9	294.2	34.2	66	9^a^	64
UG	1642.5	343.2	243.6	51.2	1673.0	357.5	182	33	177
WC	458.8	39.2	62.5	8.1	609.5	54.8	57	36	42
BC	1628.1	349.9	964.4	260.7	1850.8	379.7	225	122	166
DC2	1456.5	293.4	1451.7	321.5	1488.2	306.6	99	48	71
LM	1064.4	233.2	1081.3	255.8	864.2	170.9	262	175	182
CC^c^	NA	NA	NA	NA	NA	NA	3	3^a^	0
Mean	998.0	191.6	647.2	143.9	1025.2	192.5			
Mean^b^			760.72	179.44					

^a^ A minimum sample size of 15 (Anich et al. 2009) or 30 observations is recommended (Seaman et al. 1999).

^b^ Mean excluding sites with sample size < 30.

^c^ Site CC was used by a single female spotted owl, which was only detected over 3 days at three recording stations. No male spotted owl was detected.

## Appendix B

**TABLE B1:** Coefficient estimates (β) with standard errors (SE) and *p*‐values from quasibinomial generalized linear models (GLMs) for northern spotted owls excluding site BC, using the proportion of weeks with spotted owl territorial detections by sex (any = all territorial calls regardless of sex) as the response variable.

Model	Coefficient	Any		Female		Male	
		β (SE)	*p*	β (SE)	*p*	β (SE)	*p*
~dist	(Intercept)	0.35 (0.309)	0.25	−0.330 (0.354)	0.352	−0.188 (0.303)	0.54
	dist	−0.002 (0.0002)	< 0.01	−0.002 (0.0002)	< 0.01	−0.002 (0.0002)	< 0.01
~dist + nested	(Intercept)	0.299 (0.337)	0.38	−0.143 (0.343)	0.68	−0.006 (0.334)	0.99
	dist	−0.002 (0.0002)	< 0.01	−0.002 (0.0002)	< 0.01	−0.002 (0.0002)	< 0.01
	nested(y)	0.128 (0.302)	0.67	−1.267 (0.418)	< 0.01	0.423 (0.306)	0.168
~dist + bo_total	(Intercept)	0.799 (0.380)	0.04	0.338 (0.429)	0.43	0.631 (0.379)	0.1
	dist	−0.002 (0.0002)	< 0.01	−0.002 (0.0002)	< 0.01	−0.002 (0.0002)	< 0.01
	bo_total	−0.0004 (0.0002)	0.05	−0.001 (0.0004)	0.04	−0.0004 (0.0002)	0.06
~dist + bo_prop	(Intercept)	1.123 (0.432)	0.01	0.772 (0.458)	0.09	1.006 (0.446)	0.03
	dist	−0.002 (0.0002)	< 0.01	−0.002 (0.0002)	< 0.01	−0.002 (0.0002)	< 0.01
	bo_prop	−1.626 (0.627)	0.01	−2.425 (0.752)	< 0.01	−1.737 (0.663)	0.01

*Note:* See Table 1 for covariate definitions and Figure B1 to visualize key effects.

**TABLE B2:** Full suite of single‐species single‐season occupancy models ranked by difference in Akaike's Information Criterion (AIC) for northern spotted owls excluding site BC, including number of parameters (K), model weight (AICwt), cumulative weight (cumwt), and model structure for detection probability (*p*) and probability of use (ψ).

Sex	Model structure	K	AIC	ΔAIC	AICwt	Cumwt
Female	*p*~dist + site_year + noise + log(effort) + bo_total_wk ψ~dist + site_year	19	804.4	0.0	0.60	0.60
	*p*~dist + site_year + noise + log(effort) + bo_total_wk ψ~dist + site_year + bo_total	20	806.1	1.8	0.25	0.85
	*p*~dist + site_year + noise + log(effort) + bo_total_wk ψ~dist + site_year + bo_total + nr_prop	21	808.0	3.6	0.10	0.95
	*p*~dist + site_year + noise + log(effort) + bo_total_wk ψ~dist	13	811.8	7.5	0.02	0.97
	*p*~dist + site_year + noise + log(effort) ψ~dist	12	813.8	9.4	0.01	0.99
	*p*~dist + site_year + log(effort) ψ~dist	11	816.8	12.5	0.01	0.99
	*p*~dist + site_year + noise ψ~dist	11	832.1	27.7	0.01	1
	*p*~dist + site_year ψ~dist	10	837.4	33.1	0	1
	*p*~dist + nest ψ~dist	5	902.4	98.0	0	1
	*p*~dist ψ~dist	4	914.8	110.4	0	1
	*p*~1 ψ~1	2	994.3	189.9	0	1
Male	*p*~dist + site_year + noise + log(effort) + bo_total_wk ψ~dist + site_year	19	1323.0	0.0	0.43	0.43
	*p*~dist + site_year + log(effort) ψ~dist	11	1324.7	1.7	0.19	0.62
	*p*~dist + site_year + noise + log(effort) + bo_total_wk ψ~dist + site_year + bo_total	20	1324.7	1.7	0.18	0.80
	*p*~dist + site_year + noise + log(effort) + bo_total_wk ψ~dist + site_year + bo_total + nr_prop	21	1326.2	3.2	0.09	0.88
	*p*~dist + site_year + noise + log(effort) ψ~dist	12	1326.5	3.5	0.07	0.96
	*p*~dist + site_year + noise + log(effort) + bo_total_wk ψ ~ dist	13	1327.6	4.6	0.04	1
	*p*~dist + site_year ψ~dist	10	1345.9	22.9	0	1
	*p*~dist + site_year + noise ψ~dist	11	1347.9	24.9	0	1
	*p*~dist + nest ψ~dist	5	1428.2	105.2	0	1
	*p*~dist ψ~dist	4	1428.9	105.9	0	1
	*p*~1 ψ~1	2	1588.3	265.3	0	1
Any	*p*~dist + site_year + noise + log(effort) + bo_total_wk ψ~dist + site_year	19	1457.3	0.0	0.45	0.45
	*p*~dist + site_year + noise + log(effort) + bo_total_wk ψ~dist + site_year + bo_total	20	1458.8	1.6	0.21	0.66
	*p*~dist + site_year + log(effort) ψ~dist	11	1459.7	2.4	0.14	0.79
	*p*~dist + site_year + noise + log(effort) + bo_total_wk ψ~dist + site_year + bo_total + nr_prop	21	1459.7	2.5	0.13	0.92
	*p*~dist + site_year + noise + log(effort) ψ~dist	12	1461.5	4.3	0.05	0.98
	*p*~dist + site_year + noise + log(effort) + bo_total_wk ψ~dist	13	1463.2	5.9	0.02	1

	*p*~dist + site_year ψ~dist	10	1483.9	26.6	0	1
	*p*~dist + site_year + noise ψ~dist	11	1485.3	28.0	0	1
	*p*~dist ψ~dist	4	1598.1	140.8	0	1
	*p*~dist + nest ψ~dist	5	1600.0	142.7	0	1
*p*~1 ψ~1	2	1756.3	299.0	0	1

*Note:* Models used detections of female, male, or any sex spotted owl territorial calls collected at seven activity centers. See Table 1 in the main text for covariate definitions.

**TABLE B3:** Covariate beta coefficients (β), lower 95% confidence limit (LCL), and upper 95% confidence limit (UCL) reflecting effects on probability of use (ψ) and detection probability (*p*) for northern spotted owls using detections of female, male, or any sex territorial calls collected at seven activity centers, excluding site BC.

Group	Parameter	Covariate	β	LCL	UCL
Female	ψ	Intercept	−3.44	−5.35	−1.52
	ψ	dist	−1.30	−1.87	−0.73
	ψ	site_yearCC	0.66	−2.51	3.82
	ψ	site_yearDC	1.52	−0.68	3.72
	ψ	site_yearDC2	2.69	0.62	4.77
	ψ	site_yearLM	3.27	1.15	5.40
	ψ	site_yearUG	0.58	−1.68	2.84
	ψ	site_yearWC	0.69	−1.59	2.97
	*p*	Intercept	−17.84	−26.71	−8.96
	*p*	dist	−0.97	−1.20	−0.73
	*p*	site_yearCC	−1.20	−3.51	1.12
	*p*	site_yearDC	0.17	−1.04	1.38
	*p*	site_yearDC2	0.65	−0.52	1.83
	*p*	site_yearLM	1.48	0.24	2.72
	*p*	site_yearUG	0.40	−0.89	1.68
	*p*	site_yearWC	0.22	−1.05	1.49
	*p*	noise	−0.41	−0.74	−0.08
	*p*	log(effort)	1.24	0.50	1.99
	*p*	bo_total_wk	−0.32	−0.61	−0.02
Male	ψ	Intercept	−1.90	−3.01	−0.78
	ψ	dist	−1.13	−1.59	−0.66
	ψ	site_yearCC	−14.21	−625.73	597.31
	ψ	site_yearDC	0.38	−1.21	1.98
	ψ	site_yearDC2	1.31	−0.07	2.69
	ψ	site_yearLM	1.77	0.37	3.17
	ψ	site_yearUG	2.58	1.17	4.00
	ψ	site_yearWC	1.05	−0.58	2.68
	*p*	Intercept	−13.39	−13.79	−12.99
	*p*	dist	−0.97	−1.15	−0.80
	*p*	site_yearCC	0.20	0.20	0.20
	*p*	site_yearDC	−0.55	−1.33	0.23
	*p*	site_yearDC2	0.14	−0.55	0.82
	*p*	site_yearLM	1.04	0.31	1.76
	*p*	site_yearUG	0.99	0.37	1.61
	*p*	site_yearWC	−0.68	−1.45	0.09
	*p*	noise	0.07	−0.16	0.31
	*p*	log(effort)	0.93	0.44	1.43
	*p*	bo_total_wk	0.05	−1.52	1.63
Any	ψ	Intercept	−1.91	−3.00	−0.81
	ψ	dist	−1.11	−1.55	−0.67
	ψ	site_yearCC	0.57	−3.48	4.62
	ψ	site_yearDC	0.53	−0.97	2.04
	ψ	site_yearDC2	1.37	0.03	2.72
	ψ	site_yearLM	1.98	0.61	3.35
	ψ	site_yearUG	2.57	1.18	3.96
	ψ	site_yearWC	0.89	−0.64	2.43
	*p*	Intercept	−12.84	−18.04	−7.63
	*p*	dist	−0.96	−1.12	−0.79
	*p*	site_yearCC	−3.34	−5.27	−1.41
	*p*	site_yearDC	−0.33	−1.06	0.41
	*p*	site_yearDC2	0.46	−0.20	1.12
	*p*	site_yearLM	1.29	0.59	1.99
	*p*	site_yearUG	0.93	0.32	1.53
	*p*	site_yearWC	−0.53	−1.26	0.21
	*p*	noise	−0.01	−0.24	0.21
	*p*	log(effort)	0.89	0.46	1.33
	*p*	bo_total_wk	0.02	−0.13	0.17

*Note:* The model shown had the most support for all datasets: female, male, and any sex calls. See main text Table 1 for covariate definitions.
